# The Validity and Reliability of the Turkish Scale for the Assessment of Fatigue in Pediatric Oncology Patients Aged 7-18 in Russia

**DOI:** 10.11621/pir.2021.0104

**Published:** 2021-03-31

**Authors:** Alena A. Deviaterikova, Vladimir V. Kasatkin, Boris B. Velichovsky

**Affiliations:** a Dmitry Rogachev National Medical Research Center of Pediatric Hematology, Oncology, and Immunology, Moscow, Russia; b Russian Academy of Education, Moscow, Russia; c Lomonosov Moscow State University Faculty of Psychology, Moscow, Russia

**Keywords:** fatigue, pediatric cancer, questionnaire, adolescent, quality of life

## Abstract

**Background:**

Fatigue is the most common complaint by children both during and after cancer treatment, but in Russia, there is no reliable method for assessing fatigue.

**Objective:**

To develop a Russian version of the Turkish Scale for the Assessment of Fatigue in Pediatric Oncology Patients Ages 7-18.

**Design:**

Our first step was to translate all the items of the Turkish questionnaire into Russian. Then, through discussion, we created a single proposition for each item. The next step was obtaining expert opinions to assess the validity. Once the expert estimates agreed, a pilot version of the questionnaire was formed. The next step was to collect a large sample of patients to study the reliability and validity of the questionnaire.

**Results:**

As a result of factor analysis, three factors were identified. The first factor was "fatigue associated with actions;" the second was "fatigue as feeling;" and the third was "fatigue associated with sleep difficulties." The children's and parents’ versions had the same factor structure.

**Conclusions:**

This study showed the possibility of using the questionnaire in a Russian sample. That’s why it is necessary to continue collecting and analyzing data in this direction. The reliability of the test was also assessed. The reliability of the parent version scored a Cronbach’s alpha of 0.91. The reliability of the children’s version showed a Cronbach’s alpha of 0.93.

## Introduction

Cancer is a serious disease that occurs throughout the world (Stewart & Wild, 2014; Jemal, 2011; Stewart et al., 2019). It affects both children and adults. In addition to the pain of the disease and treatment, patients suffer emotional and behavioral disorders (Olson et al., 2008).

The most common cancer complaint is fatigue (Curt et al., 2000). It is the most distressing symptom for patients, since it reduces their quality of life (Bower et al., 2000).However, patients who have completed treatment and are in remission also have symptoms of fatigue. Even after their cancer is cured, patients have chronic illnesses due to their treatment (Oeffinger et al., 2006). Fatigue remains with some patients for many years after the completion of treatment (Bower et al., 2006). Many studies show that over 50% of adults treated for cancer have signs of fatigue after finishing treatment (Richardson et al., 1998). Adult fatigue has psychological, physiological, and emotional components (Richardson et al., 1998; Akechi et al., 1999).

The chronic fatigue syndrome of cancer survivors has not been studied. In the first attempt at such a study, a survey was conducted of children with cancer, their parents, and the hospital staff who worked with the children. There was children’s group of 7-12 year-olds and one of 13-18 year-olds (Winningham et al., 1994). The children complained of fatigue and the lack of enough strength to even get up and open their eyes. They had no desire to play and learn. They wanted to lie down and do nothing, and their parents allowed them not to attend classes and school, because the children complained of fatigue as soon as they woke up.

The task of a psychologist who wants to help such children is to help them cope with fatigue (Hockenberry-Eaton et al., 1998). To do this, however, you should first determine the degree of fatigue. Fatigue can be alleviated, and therapy can improve patients' quality of life (Varni et al., 2005). In order to alleviate the symptoms of fatigue in children, joint work by the therapist, the parents, and the hospital staff is necessary (Miaskowski, 2006). The feeling of fatigue prevents children from leading active lives, as well as preventing them from getting an education. Children can be cognitivelyintact, but lack of strength does not allow them to effectively solve problems. High degrees of fatigue prevent children from attending school, which leads to emotional deprivation and a decrease in social contact. In addition, the quality of home education may be lower than that at school (Wu et al., 2010).

Therefore, fatigue is a serious problem for cancer survivors. Since it affects the quality of a patient’s life, it’s necessary to diagnose it as quickly as possible.

In order to assess fatigue, a reliable method is required. However, in Russia there is no such method for assessing fatigue in children who have survived cancer. Current methods for assessing fatigue are not reliable and valid (Miaskowski, 2006; Wu et al., 2010). Most often, if a child complains of fatigue, he is permitted more rest and freed from any activities.

In world practice, however, fatigue is assessed using questionnaires. For example, in Turkey there is a national questionnaire for assessing fatigue. The Turkish Chronic Fatigue Questionnaire was developed for children 7-12 and 13-18 years of age, and their parents. This questionnaire has shown its reliability, validity, and consistency in Turkish samples (Gerceker & Yilmaz, 2012; Kudubes et al., 2014; Bektas & Kudubes, 2014).

The aim of our study was to adapt the Turkish questionnaire measuring the Scale for the Assessment of Fatigue in Pediatric Oncology Patients Aged 7-12 and 13-18, for use with a Russian population.

## Methods

### Participants

Our sample included 295 children (*Table 1*). The mean age of their oncology diagnoses was 7.25 years (SD = 1.5 years). Their periods of remission ranged from 24 to 72 months, with most children having received that status by the age of 8.2. The older the child, the longer the remission period (for example, for a 12 year-old child’s remission period was 3.8 years; for 15 year-olds, ; 6.8 years). Mothers filled out the parent version of the questionnaire.

**Table 1:** Description of Sample

Patients	N	M (SD)	% boys
Medulloblastoma	95	12.1 (3.0)	53
Acute lymphoblastic leukemia	50	12.07 (2.7)	45
Hodgkin's lymphoma	50	11.5 ;(3.2)	40
Hematopoietic stem cell transplantation	50	10.95 ;(3.04)	39

Parents and children over 14 signed informed consent forms to participate in the study. An ID number was assigned to each patient to maintain anonymity. The children spent from seven to 32 days as patients at a rehabilitation center, and received various forms of rehabilitation depending on their needs. Some children underwent motor training and some cognitive training; some children saw a psychotherapist (individually or in a group). Also, all the children underwent physiotherapy, which included swimming. During admission to the center, the children received an invitation to participate in the study assessing chronic fatigue syndrome. So, their responses were not affected by their presence in the center. Procedures can be difficult for a child and increase his fatigue.

### Measures

The original version of the Scale for the Assessment of Fatigue in Pediatric Oncology Patients had been developed in Turkey and consisted of a total of 27 items and 3 factors. The factor of general problems was measured by the first eighteen items. This factor measured overall fatigue. The next factor measured fatigue associated with difficulty sleeping (9-24 items). The last factor consisted of 25-27 items and evaluated fatigue during treatment procedures. We carried out the following stages of the Scale for the Assessment of Fatigue in Pediatric Oncology Patients (Gerceker et al., 2012; Kudubes et al., 2014; Bektas & Kudubes, 2014).

We used the scales presented by Aslı Akdeniz Kudubes (Gerceker et al., 2012; Kudubes et al., 2014; Bektas & Kudubes, 2014). The first step was to translate all the items of the questionnaire into Russian. Eight psychologists, five doctors, two biologists, and one translator took part. Each expert presented his version of the translation. Then, through discussion, they created a single proposition for each item. This stage lasted a month and a half. The result was a list of items in Russian.

The next step was an expert assessment, which took place in several stages. The pool of translated items was sent to several independent experts for improving the translation. The aim was to get an identical interpretation of the items after reverse translation.

The experts studied the translated items and evaluated how each statement related to what we wanted to measure, and whether this affirmation was clear to the respondents. If the experts’ assessments had significant differences, the additional opinion of other experts in this field was required.

Then, 16 expert opinions were obtained to assess the validity of the content of the scales (Bektas & Kudubes, 2014; Akgul, 2003; Gozum & Aksayan, 2002; Ozdamar, 2005; Sencan, 2005).

Once the expert estimates were agreed upon, a pilot version of the questionnaire was created. Respondents were to grade each item on a Likert scale. The pilot version of the test was offered to 20 patients. This was necessary in order to assess how clear the items were for the patients. At this stage, we also checked whether the patients understood the items correctly. Both parents and children filled out the questionnaires and affirmed that all the items were understandable and did not have different interpretations. Then, the next step was to collect a large sample of patients to study the reliability and validity of the questionnaire.

To assess thecoherencerelationship of our questionnaire with other valid questionnaires, we used the Achenbach Children Behavior Checklist for assessing emotional states. The Achenbach Children Behavior Checklist (CBCL) has three forms: one for parents, one for children, and one for teachers. In this study, we used only the ones for parents and children. The questionnaire was standardized in Russian, and described behavioral and emotional problems.

## Results

### Construct Validity

The results of the assessment by the 16 experts were tested for accuracy. The consistency scores were determined to be 0.716. Thus, the estimates were considered consistent.

*The Parent Form of the Scale for the Assessment of Fatigue in Pediatric Oncology Patients*: Construct validity of the scales was tested by factor analysis, which showed the Kaiser-Meyer-Olkin coefficient (KMO) to be 0.931. The resulting factor structure differed from the original. In our study, three factors were also highlighted, but the distribution of assertions varied.

The first factor can be called "fatigue associated with actions" (it included items 2, 8, 9, 10, 11, 12, 13, 15, 17, 25, 26, & 27);the factor loads were determined to be 0.42-0.68. The second factor was "fatigue as feeling" (it included items 1, 4, 5, 7,14, 16, 18, & 22); the factor loads were determined to be 0.42-0.72. The third factor was "fatigue associated with sleep difficulties" (it included items 3, 6, 19, 20, 21, 23, & 24); the factor loads were determined to be 0.48-0.66.

The "fatigue associated with actions" explained 38.3% of the total variance; the "fatigue as feeling" explained 38.3%; and the "fatigue associated with sleep difficulties" explained 18.9%. The total of the three explained 94% (see *Table 2* in the Appendix).

*The Child Form of Scale for the Assessment of Fatigue in Pediatric Oncology Patients*: As a result of the factor analysis, the Kaiser-Meyer-Olkin coefficient (KMO) was determined to be 0.888. The resulting factor structure differed from the original. In our study, three factors were also highlighted, but the distribution of assertions varied.

The first factor can be called "fatigue associated with actions" (it included items 2, 8, 9, 10, 11, 12, 13, 15, 17, 25, 26, & 27);the factor loads were determined to be 0.40-0.75. The second factor was "fatigue as feeling" (it included items 1, 4, 5, 7,14, 16, 18, & 22); the factor loads were determined to be 0.46-0.68. The third factor was "fatigue associated with sleep difficulties" ( it included items 3, 6, 19, 20, 21, 23, & 24); the factor loads were determined to be 0.46-0.68.

The "fatigue associated with actions" explained 33.4% of the total variance; the "fatigue as feeling" explained 33.4%; and the "fatigue associated with sleep difficulties" explainsed 18.9%. The total variance explained by the factors was 82.9% (See *Table 3* in the Appendix).

#### Internal consistency analysis

One of the steps required to successfully adapt the questionnaire was to evaluate its internal consistency; that is, how much did each item in the test correspond to what the questionnaire measured? The reliability coefficients of the factors of the parent form showed a Cronbach’s alpha of 0.91. The reliability coefficients of the factors of the child form showed a Cronbach’s alpha of 0.93.

#### Relationship to other questionnaires

The coherence between the parent version of the questionnaire Scale for the Assessment of Fatigue in Pediatric Oncology Patients and the parent version of the Achenbach Child Behavior Checklist was evaluated; it amounted to p = 0.001. The relationship between the child version of the Scale for the Assessment of Fatigue in Pediatric Oncology Patients and child form of the Achenbach Child Behavior Checklist measured p = 0.001.

## Discussion

In the present study, we completed the first stage of the adaptationof the Turkishquestionnaire Scale for the Assessment of Fatigue in Pediatric Oncology Patients for use in Russia. We showed that this questionnaire had validity and reliability.Weassessed the validity of the questionnaire:it was translated with the help of experts from various fields who work with this cohort of patients. We also obtained expert opinions of psychologists. As a result, we created the first version of the questionnaire, which was used on for getting a large sample of patients.

Fatigue is a common complaint of cancer survivors.Fatigue prevents children from leading a standard lifestyle, communicating with peers, and attending educational institutions. Often, due to fatigue, children cannot cope with cognitive tasks. In this case, they don’t have problems with cognitive functions, but they lack enough strength to solve cognitive tasks. Since fatigue is an obstacle to a full life, high-quality tools are needed to evaluate it.

We then carried out a factor and explanatory analysis. The goal of the factor analysis was to identify the structure of the relationship between the variables and highlight the factors describing them (Gozum & Aksayan, 2002; Ozdamar, 2005; Simsek, 2007). In the course of a factor analysis, from the first rotation, the varimax structure was divided into three factors.The first factor was "fatigue associated with actions;" the second was "fatigue as feeling;” and the third was "fatigue associated with sleep difficulties." The resulting factor structure differed from the original. In our study, three factors were also highlighted, but the distribution of assertions was different.

Currently, in Russia, rehabilitation programs for cancer survivors are just beginning to be created. These programs are based on correction of the children’s cognitive and motor deficits, but don’t take into account the emotional consequences of cancer. However, fatigue complaints are the most frequent. Adaptation of the Turkish tool will help professionals to assess the degree of fatigue and create a rehabilitation plan for each child. This study showed the possibility of using the Turkish questionnaire with the Russian sample. It is necessary to continue collecting and analyzing data in this direction.

## Conclusion

As a result of our study, a questionnaire was created which assesses chronic fatigue syndrome in Russian children who survived cancer. This questionnaire showed high validity and reliability in the Russian sample. The advantage of this questionnaire is the presence of both a children's and parental version, because the subjective feelings of the child and the parent may differ; having both versions will be helpful for the doctor’s diagnosis. While our sample was small, this study was only the first step toward collecting extensive data. The questionnaire will be distributed to other children's centers in Russia for more extensive data collection. Our research has not yet presented data on test-retest reliability; this is the subject of further research.

## Limitations

There are possible issues with the ability to generalize the results,*e.g*., sample size and limited access to data.

One limitation is that the sample size was not very large. Another limitation is that the sample was not divided by oncological diagnoses. Additional research is needed on large samples of children with various diagnoses and different ages.

In the creation of the Russian version of the questionnaire, only a direct translation of the questionnaire into Russian was carried out. No back translation from Russian was made. Also, test-retest reliability was not evaluated.

## Appendix

**Table 2:** Factor structure of the Parent Form of the questionnaire

Factor name	Items included in factor	Factor loads
Fatigue associated with actions	He/She feels tired even if eating	0.544
	He/She needs to stop and rest while walking	0.466
	He/She needs help in doing his/her daily work	0.482
	He/She feels powerless for do his/her favorite things (play games, spend time with his/her friends, etc.)	0.515
	He/She is having trouble starting his/her day job.	0.42
	He/She is having trouble finishing his/her daily business	0.417
	He/She needs to rest too much	0.424
	He/She feels too tired to deal with his/her external appearance	0.422
	He/She feels sick	0.459
	He/She feels tired before treatment	0.487
	He/She feels tired during treatment	0.512
	He/She feels tired after treatment	0.574
Fatigue as feeling	He/She feels tired	0.507
	He/She feels more tired in the afternoon	0.456
	He/She feels more tired in the evening	0.42
	He/She wants to just lie down and rest	0.485
	He/She doesn’t want to do anything	0.476
	He/She feels exhausted/sluggish	0.518
	He/She has had to deal with fatigue during the day	0.439
	He/She needs to sleep during the day (nap)	0.43
Fatigue associated with sleep difficulties	He/She feels more tired in the morning	0.491
	He/She has trouble getting out of bed during the day	0.432
	He/She wakes up tired in the morning	0.412
	He/She sleeps too much	0.568
	He/She is having trouble keeping his/her eyes open	0.655
	He/She has trouble falling asleep at night	0.535

**Table 3:** Factor structure of the Child Form of the questionnaire

Factor name	Items included in factor	Factor loads
Fatigue associated with actions	I feel tired even if eating	0.617
	I need to stop and rest while walking	0.553
	I am able to do my usual activities	0.576
	I have felt angry	0.576
	I have not felt like talking	0.469
	I need help to do my usual activities	0.458
	I don’t feel like being with others	0.431
	I feel too tired to deal with my external appearance	0.427
	I feel sick	0.434
	I feel tired before treatment	0.606
	I feel tired during treatment	0.55
	I feel tired after treatment	0.597
Fatigue as feeling	My body has felt tired	0.606
	I want to rest more	0.51
	I sleep more often	0.462
	I don’t feel like doing much	0.595
	I don’t want to do anything	0.574
	I feel exhausted/sluggish	0.632
	I have had to deal with fatigue during the day	0.536
	I need to sleep during the day (nap)	0.48
Fatigue associated with sleep difficulties	I feel more tired in the morning	0.535
	I have trouble getting out of bed during the day	0.414
	I sleep too much	0.493
	I wake up at night consistently	0.362
	I wake up tired in the morning	0.568
	I have trouble keeping my eyes open	0.604
	I have trouble falling asleep at night	0.579
